# Immune Protection of a Helminth Protein in the DSS-Induced Colitis Model in Mice

**DOI:** 10.3389/fimmu.2021.664998

**Published:** 2021-04-29

**Authors:** Shao Rong Long, Ruo Dan Liu, Deepak Vijaya Kumar, Zhong Quan Wang, Chien-Wen Su

**Affiliations:** ^1^ Department of Parasitology, Medical College of Zhengzhou University, Zhengzhou, China; ^2^ Mucosal Immunology and Biology Research Center, Massachusetts General Hospital and Harvard Medical School, Charlestown, MA, United States

**Keywords:** *Trichinella spiralis*, colitis, dextran sodium sulfate, macrophage, immune protection

## Abstract

Inflammatory bowel disease (IBD) increases the risk of colorectal cancer, and it has the potential to diminish the quality of life. Recent clinical and experimental evidence demonstrate protective aspects of parasitic helminth infection against IBD. Reports have highlighted the potential use of helminths and their byproducts as potential treatment for IBD. In the current study, we studied the effect of a newborn larvae-specific serine protease from *Trichinella spiralis* (TsSp) on the host immune and inflammatory responses. A 49-kDa recombinant TsSp (rTsSp) was expressed in *Escherichia coli* BL21 (DE3) and purified. The cytotoxicity of rTsSp was analyzed. The immune protective effect of rTsSp was studied by using dextran sodium sulfate (DSS)-induced mouse colitis model. The result illustrated that rTsSp has no toxic effects on cells. We further demonstrated that administration of the rTsSp without the additional adjuvant before the induction of DSS-induced colitis reduced the severity of intestinal inflammation and the disease index; it suppressed macrophage infiltration, reduced TNF-α secretion, and induced IL-10 expression. Our findings suggest therapeutic potential of rTsSp on colitis by altering the effect of macrophages. Data also suggest immunotherapy with rTsSp holds promise for use as an additional strategy to positively modulate inflammatory processes involved in IBD.

## Introduction

Inflammatory bowel disease (IBD) is characterized by mucosal damage and ulceration that encompasses Crohn’s disease (CD) and ulcerative colitis (UC) (1). The increasing prevalence of IBD will likely diminish quality of life and increase the risk of colorectal cancer (2, 3). IBD inflammation results from a complex innate and adaptive cytokine network (1). Although the exact cause of IBD remains unknown, past investigations have suggested that the pathogenesis of IBD involve genetic factors, changes in the gut microbiome, and immune pathways including cytokines and immune cells (4).

Evidence from epidemiological studies of the IBD hygiene hypothesis indicate that helminth infections have modulatory effects in certain immune-mediated diseases, including IBD (5). Helminths, characterized by their ability to maintain long-standing, chronic infections in humans, have infected more than 2 billion people worldwide (6).

Helminth infections lead to modulated immune responses with a strong Th2 bias and a major immunoregulatory component, involving the secretion of interleukin (IL)-4, IL-5, IL-9, IL-10 and IL-13, and accompanied by the hyperplasia of mast cells, eosinophils, goblet cells and innate lymphoid cells (ILCs) (7, 8). Also involved is the expansion of both peripheral and thymic regulatory T cells (Tregs) (9). The regulation of parasite-specific immune response affects not only helminth antigens, but also unrelated antigens. In recent years, clinical and experimental evidence has demonstrated that infection with parasitic helminth protects hosts from IBD; for example, a case control study in South Africa demonstrated protective association of childhood helminth infection against the development of IBD (10)**;** pre-existing helminth *Echinococcus multilocularis* (11), *Heligmosomoides polygyrus* (12), or *Hymenolepis diminuta* (13), etc., infection have been shown to reduce chemically-induced colitis in mice by modulating the immune responses of their hosts.

The helminth *Trichinella* has a unique life cycle: unlike other helminths, all stages in the life cycle of larval and adult occur in the same host (14). The immunomodulatory properties of *T. spiralis* have been validated in several models of immunopathology, and experiments have shown that *Trichinella* infection can ameliorate some immuno-mediated disease pathology. For example, *T. spiralis* infection ameliorates experimental dextran sodium sulfate (DSS)-induced colitis in mice. The amelioration results from downregulation of pro-inflammatory cytokines- IL-6 and interferon gamma (IFN-γ) and upregulation of regulatory cytokines- IL-10 and transforming growth factor (TGF)-β by spleen lymphocytes and mesenteric lymph nodes (MLN). This is also accompanied by increased CD4^+^CD25^+^Foxp3^+^ Treg cell recruitment in spleen lymphocytes and MLN (15). Moreover, the effects of Excretion–Secretion products released by *Trichinella* in the modulation of experimental colitis have also been described (16).

Although parasitic helminthes and helminth products are regarded as potential therapy for autoimmune and inflammatory disorders in humans (17, 18), treatment with live worms is not a very attractive notion. Unanswered questions regarding the use of helminths in intestinal disease, such as appropriate dosing regimens, optimal timing of treatment, the role of host genetics, diet and environment warrant investigation (18). Moreover, helminth products are composed of a complex mixture of molecules, so it is still unclear on exact mechanisms of their protective effects. Therefore, the individual molecules responsible for the protective effects are to be identified to address these questions. Early study has shown that infection with *T. spiralis* resulted in the attenuation of experimental colitis in mice (19). One superfamily of enzymatic proteins, serine proteases (SPs), has been found in all stages of the *T. spiralis* (20). From the previous studies, we know that larvae-specific serine protease of TsSp has a key immunodominant region involved in protective immunity against *Trichinella* infection in mice by promoting a balance between types 1 and 2 immune responses (21, 22). Here, we examined the effect of treating the female C57BL/6 mice with recombinant *T. spiralis* newborn larvae-specific Serine protease (rTsSp) on experimentally DSS-induced colitis. Among various chemical induced colitis models, DSS-induced colitis model is widely used because of its simplicity and many similarities with human UC (23). We hypothesized that immunization with *T. spiralis* derived protein-rTsSp could induce an immuno-regulatory response that reduces the development of colitis in the mice gut.

In the present study, we provide evidence that administration of rTsSp without the addition of adjuvant before the induction of colitis with DSS reduces the severity of intestinal inflammation and the disease index, and results in modulation of macrophage responses associated with regulatory cytokine expression. These observations suggest that the unique helminth derived protein rTsSp is able to ameliorate disease signs in an acute model of colitis *via* its immunomodulatory properties.

## Materials and Methods

### Mice

Pathogen-free, 8-week-old female C57BL/6 mice were purchased from the Experimental Animal Center of Henan Province. They were fed autoclaved food and water. The Institutional Subcommittee on Research Animal Care approved all animal experiments.

### Preparation of rTsSp Protein

The *TsSp-* pET-22b (+) recombinant plasmid was provided by Prof. Mingyuan Liu from Jilin University. Recombinant proteins (rTsSp) were expressed in *Escherichia coli* BL21 (DE3), purified with Ni-NTA Spin Kit (QIAGEN, Catalog No. 31314), and analyzed with the sodium dodecyl sulfate–poly acrylamide gel electrophoresis (SDS-PAGE) as previous studies (24, 25) and silver staining as the manufacturer’s instruction (Sigma, Product Code PROT-SIL1). The rTsSp concentration was detected by using BCA Protein Assay Reagent (Thermo Fisher Scientific, Catalog No. 23223) and Synergy 2 (Bio-Rad).

### Cell Viability Testing With Trypan Blue Exclusion

Prior to incubation, Raw 264.7 cells were seeded at 10^6^ cells/ml in 12-well cell culture plates overnight. Cells were washed twice with PBS and incubated with 5 μg/ml, 10 μg/ml, and 20 μg/ml rTsSp for 6, 12, and 24 h. On the indicated time, cells were collected. Cell count and viability measurements using trypan blue stain (0.4%, Gibco, REF# 15250-061) were performed by Countess Automated Cell Counter (Invitrogen).

### FAM-FLICA *In Vitro* Caspase Detection

To differentiate between apoptotic and necrotic cells, we used carboxyfluorescein (FAM)- fluorochrome-labeled inhibitors of caspases (FLICA) *in vitro* Caspase Detection Kit (ImmunoChemistry Technologies’, Catalog # 92) to assess cell death *in vitro*. Cells labeled with FAM-FLICA can be counter-stained with the red live/dead stains Propidium Iodide (PI, included in FAM-FLICA kits) to distinguish apoptosis from necrosis. Cells at 5 × 10^5^ cells/mL per sample were used for FLICA-PI staining following the manufacturer’s recommendations. 30× FLICA were added at a ratio of 1:30. Cells were incubated at 37°C protected from light for 2 h and then washed by 1 mL 1× apoptosis wash buffer (included in FAM-FLICA kits). To avoid cells from settling on the bottom of the tubes, the tubes were gently resuspended by swirling cells every 20 min to ensure an even distribution of FLICA. The samples were centrifuged at 1200 rpm for 5 min at room temperature to pellet cells, supernatants were aspirated. Cell pellets were resuspended with 0.5% v/v of PI and incubated at RT protected from light for 5 min, then washed two times by 1× apoptosis wash buffer and fixed by Fixative (included in FAM-FLICA kits) at a ratio of 1:10. The stained samples were analyzed with a BD FACSCanto. Debris was excluded from analysis *via* size exclusion. Four populations of cells were revealed: cells in early apoptosis fluoresce green with FAM-FLICA, cells in late apoptosis are dually stained with FAM-FLICA and PI, necrotic cells fluoresce red and unstained live cells have minimal fluorescence.

### *In vitro* Stimulation of Raw 264.7 Cells

Raw 264.7 cells were maintained at 37°C in 5% CO_2_ in cDMEM. The cells were seeded at 10^6^ cells/ml in 24-well cell culture plates. Cells were pre-treated with/without rTsSp 1 day before or at the same time when stimulated with LPS. After 5/10 hours stimulation, the supernatants were collected, and the levels of TNF-α present were detected *via* ELISA.

### Immunization Schedule and DSS-Induced Colitis

**Figure 1:**
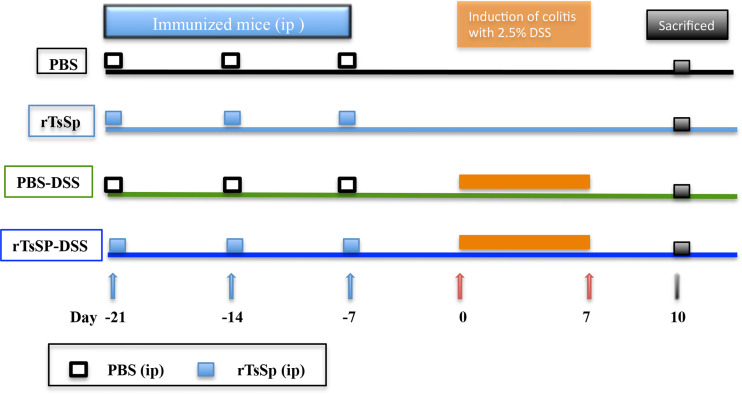
Immunization and DSS-induced colitis schedule.

C57BL/6 mice were divided into four groups (1. PBS; 2. PBS-DSS; 3. rTsSp; 4. rTsSp-DSS), immunized three times with intraperitoneal injection at weekly intervals between each injection; three independent experiments were performed (5 mice per group). Groups 3 and 4 were immunized with 50 μg rTsSp in 100 μl phosphate-buffered saline (PBS) without any adjuvant. Groups 1 and 2 were immunized with 100 μl PBS as control. The last injection was performed 1 week before DSS treatment. About 50 μl of tail blood of immunized mice were collected at day 0. The levels of the specific antibodies in serum were determined by ELISA as previous studies (24). Experimental colitis was induced in groups 2 and 4 by administering a 2.5% (w/v) solution of DSS (M.W. 36000–50000 kDa; MP Biomedicals, Cat. no. 106110) to mice as a substitute for autoclaved water for 7 days. Mice were monitored daily for clinical signs including weight loss, piloerection, mobility, and fecal consistency/bleeding. At day 7 post DSS administration, the 2.5% DSS solution was replaced by autoclaved water. After additional 3 days of autoclaved water administration, the mice were sacrificed and samples were obtained and processed (**Figure 1**).

### Colon Collection

The colon tissues (from cecum to rectum) were separated after sacrificing mice. Colon length was recorded, a 0.5- to 1-cm colon piece (cecum side) was collected for the measurement of tissue cytokine expression by using real-time qPCR, the rest of the colon tissue was cut longitudinally and rolled with a toothpick for histological assessment of inflammatory infiltration.

### Histopathological Analysis

At necropsy, colon tissues were collected, formalin-fixed and embedded in paraffin. The process tissues was sectioned into 5-μm-thick slices and stained with hematoxylin and eosin (H&E) to assess the pathology.

### Immunohistochemical Analysis

The slides were blocked with avidin/biotin agent (Vector Laboratories). To analyze the abundance of infiltrating cells, colon slides were stained with FITC-labeled anti-mouse CD11b (Biolegen, 1:200). Nucleus were stained and mounted using the 4′, 6-diamidino-2-phenylindole (DAPI) (Vector Laboratories). Sections were analyzed by microscopy and the quantification was performed by ImageJ.

### *In Vitro* Stimulation of Peritoneal Macrophages

Peritoneal macrophages were isolated from normal and rTsSp-immunized mice by incubating total peritoneal cells in complete Dulbecco’s modified Eagle’s medium (cDMEM contains 10% FBS, 2 mM l-glutamine, 100 U/ml penicillin, 100 μg/ml streptomycin, 0.1 mM nonessential amino acids, and 1 mM sodium pyruvate) at 37°C for 2 h. Non-adherent cells were removed by washing with PBS, the adherent macrophages were treated with lipopolysaccharides (LPS, 100 ng/ml, Sigma), PBS was used as control. After 5 h, supernatants were collected and analyzed for the levels of TNF-α present *via* enzyme-linked immunosorbent assay (ELISA).

### 2′,7′-Dichlorofluorescin Diacetate Intracellular ROS Detection Assay

Intracellular Reactive oxygen species (ROS) generation was measured using 2′,7′-dichlorofluorescin diacetate (DCFDA) Cellular ROS Detection Assay Kit (Abcam, Ab113851) in accordance with the manufacturer’s instructions. Peritoneal cells were collected by washing the peritoneal cavity of wild type (WT) or rTsSp pre immunized mice with ice-cold PBS. First, Peritoneal macrophages were identified with anti-F4/80 (eBioscience) and CD11b (abcom) monoclonal antibody (mAb). Subsequently, cells were incubated with 200 nM phorbol 12-myristate 13-acetate (PMA; Sigma-Aldrich) and 20 μM DCFDA at 37°C for 30 min. Cells without PMA treatment were used as controls. The stained samples were analyzed with a BD FACSCanto. Dead cells and debris were excluded from analysis *via* size exclusion. Results are expressed as fold change of mean fluorescent intensity after background subtraction between control and treated cells. Each experiment was performed in triplicate.

### Real-Time qPCR Analysis

Total RNA prepared from colon tissues was reverse transcribed into cDNA. cDNA was synthesized by using 1 μg total RNA. The cDNA samples were then tested for the expression of *IL-10* (F, CCACAAAGCAGCCTTGCA; R, AGTAAGAGCAGGCAGCATAGCA) and *TNF-α* (F, CCCTCACACTCAGATCATCTTCT; R, GCTACGACGTGGGCTACAG) by real-time quantitative PCR using SYBR Green PCR Master Mix (Apex Bioresearch Product) on a 7500 Fast Real-Time PCR system (Applied Biosystems). *GAPDH* (F, GGTTGTCTCCTGCGACTTCA; R, TGGTCCAGGG TTTCTTACTCC) was used as the housekeeping control. The gene expression level was normalized by subtracting the expression level of GAPDH of the same group, and the different expression levels were calculated using the comparative Ct (2^-ΔΔCt^) method.

### Statistical Analysis

All results were expressed as the mean ± SEM. Statistical differences were determined using a two-tailed Student t test or One-way ANOVA with SPSS 21.0 software. A *P* value <0.05 was considered significant.

## Results

### Expression and Identification of rTsSp Protein

**Figure 2:**
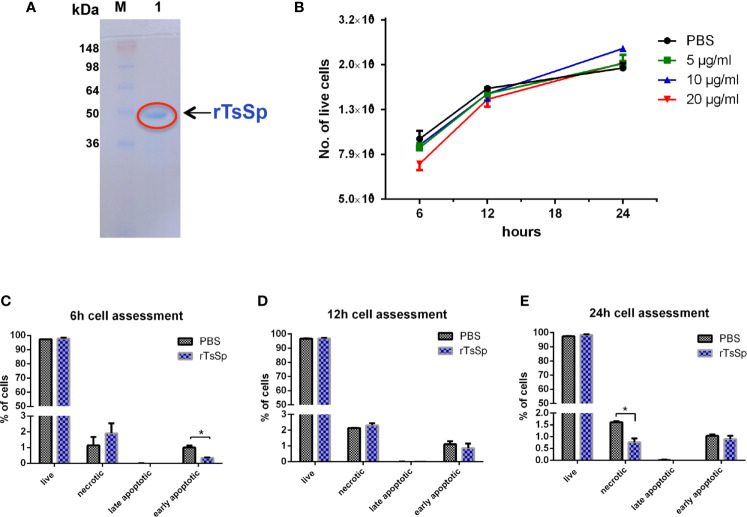
Cytotoxicity assay for the rTsSp. **(A)** SDS-PAGE analysis of rTsSp protein. M: protein molecular weight marker; 1: rTsSp purified by Ni-NTA Spin Kit. **(B)** Cell viability testing with trypan blue exclusion: Raw264.7 cells were treated with different concentrations of the rTsSp in different time points, then collected and analyzed using Countess Automated Cell Counter. **(C–E)** FAM-FLICA *in vitro* caspase detection: Raw264.7 cells were treated with 10 μg/ml rTsSp in different time points, then collected and stained with fluorochrome-labeled inhibitors of caspases (FLICA) and Propidium Iodide (PI). Stained Cells were analyzed on the BD FACSCanto. Four populations of cells were detected: FLICA^-^PI^-^ indicated unstained live cells; FLICA^-^PI^+^ necrotic cells; FLICA^+^PI^+^ cells in late apoptosis; FLICA^+^PI^-^ cells in early apoptosis. The data shown are means ± SEM from three separate experiments. *P < 0.05.

The cDNA encoding full-length TsSp is 1600 bp, encodes 430 amino acids with a predicted molecular weight of 49 kDa. Recombinant proteins expressed in *Escherichia coli* BL21 (DE3) and purified using Ni-NTA Spin Kit. The SDS-PAGE and silver staining (**Supplementary Figure 1**) analysis showed that an individual protein band with a molecular weight around 49 kDa was observed (**Figure 2A**) indicating the recombinant TsSp (rTsSp).

### Cytotoxicity Assay Illustrated that rTsSp has no Toxic Effects on Cells

Trypan blue is a stain that leaves nonviable cells with a distinctive blue color, while viable cells appear unstained. To determine if there was any cytotoxicity from rTsSp, we did cell viability testing with trypan blue exclusion. Raw 264.7 cells were incubated with 5 μg/ml, 10 μg/ml, and 20 μg/ml rTsSp for 6, 12, and 24 h. On the indicated time, cells were collected and the number of the live cells were counted. Results showed live cells increased with time in both the absence and presence of rTsSp, thus indicating that rTsSp had no influence on the cell viability (**Figure 2B**). The cell viability testing with trypan blue exclusion is a method that tests the membrane integrity of the cells, it cannot differentiate whether the cells are truly nonviable or just damaged a little bit. To differentiate between apoptotic and necrotic cells, we used FAM-FLICA *in vitro* Caspase Detection Kit to assess cell death by detecting apoptosis *in vitro*. Cells labeled with FAM-FLICA can be counter-stained with the red live/dead stains Propidium Iodide (PI) to distinguish apoptosis from necrosis. The fluorescence-activated cell sorting (FACS) data showed that the percent of early apoptosis cells was decreased in cells incubated with rTsSp when compared to the control cells at the time point of 6 h (**Figure 2C**), and the percent of necrotic cells decreased in cells incubated with rTsSp in a statistically significant manner when compared to control cells at the time point of 24 h (**Figure 2D**). However, the overall percent of unstained live cells in different time points did not appear to be either diminished or amplified in cells incubated with rTsSp when compared to control cells incubated with PBS (**Figures 2C–E**). These results demonstrated that rTsSp had no toxic effects on cells, in fact, it suppressed early apoptosis and necrosis.

### rTsSp Suppresses LPS-induced TNF-α Production by Raw 264.7 Cells

**Figure 3:**
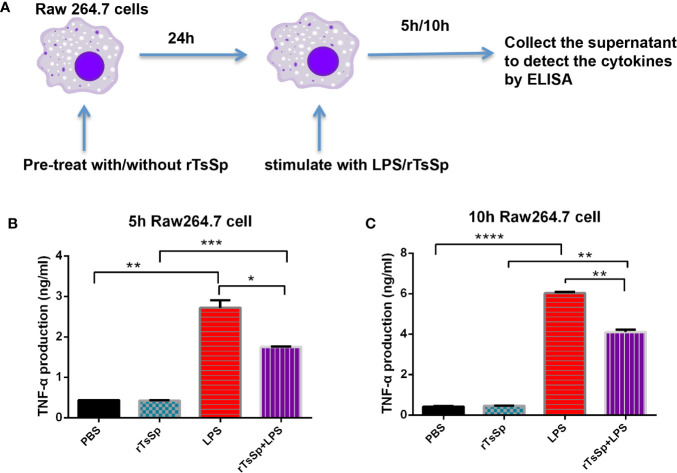
rTsSp reduced TNF-α production by macrophages. **(A)** Raw 264.7 cells were pre-treated with/without rTsSp 1 day before or at the same time when stimulated with LPS; after 5 or 10 h stimulated, the supernatant was collected; the TNF-α production of the supernatants of **(B)** 5 h and **(C)** 10 h were detected by ELISA. The data shown are means ± SEM from one of three separate experiments performed showing similar results. *P < 0.05, **P < 0.01, ***P < 0.001, ****P < 0.0001.

To analyze the effect of rTsSp on macrophage responses, the TNF-α production from Raw 264.7 cells was evaluated *in vitro*. Specifically, Raw 264.7 cells were pre-treated with/without rTsSp 1 day before or at the same time when the cells were stimulated with LPS. After 5- or 10-h stimulation, the supernatants were collected to detect the cytokine by ELISA (**Figure 3A**). Results showed that LPS-treatment induced a marked increase in macrophage TNF-α production. A significant reduction of LPS-induced TNF-α production was detected in cells that were pre-treated with rTsSp. Cells treated with rTsSp alone did not induce TNF-α production (**Figures 3B, C**).

### *Trichinella-*derived Protein (rTsSp) Suppresses Clinical Signs in DSS-Induced IBD

**Figure 4:**
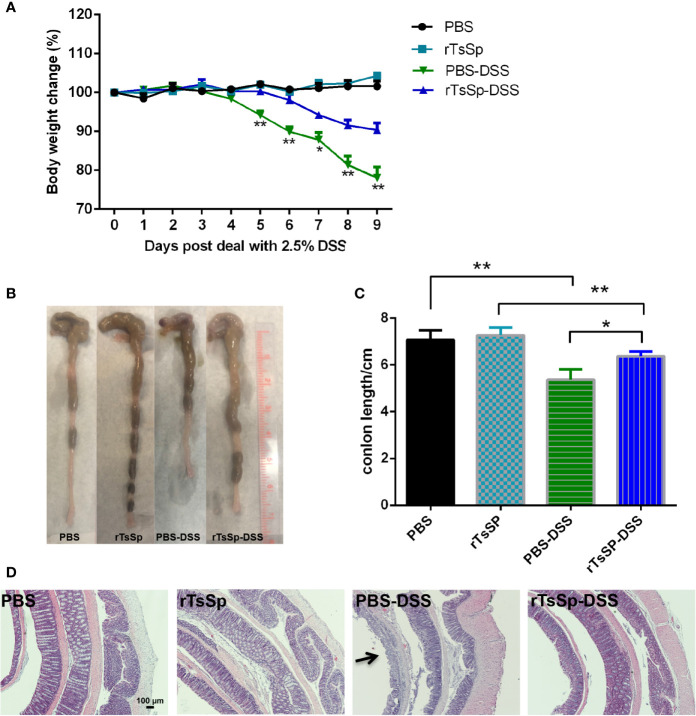
Immunization of the unique *Trichinella*-derived protein-rTsSp prevents DSS-induced colitis in mice. **(A)** Weight change in percent, **(B)** macroscopic characteristics of the colon, **(C)** colon length and **(D)** H&E stain of colonic sections were evaluated. The data shown are means ± SEM (5 mice per group) from one of three separate experiments performed showing similar results. *P < 0.05, **P < 0.01.

DSS acute colitis model was used to assess whether rTsSp was able to produce the modulation of intestinal inflammation. The mice were immunized with rTsSp three times, prior to DSS administration. Anti-rTsSP specific antibody levels in mice immunized with rTsSP increased following the immunization (**Supplementary Figure 2**). As expected, the DSS-positive control group exhibited visible signs of inflammation characterized by a hunched back, weight loss, piloerection, inactivity, and displayed important macroscopic and histological lesions, while pre-administration of rTsSp led to a significant decrease in weight loss and disease symptoms in DSS colitis. PBS and rTsSp-immunized mice showed no weight loss or clinical disease symptoms. Mice treated with DSS showed weight loss starting on day 5 after DSS administration; previous immunization with rTsSp showed weight loss starting on day 7 after DSS administration, and resulted in a statistically relevant decrease in body weight loss when compared to DSS positive control group (**Figure 4A**). The body weights of mice in the PBS control and rTsSp immunization alone groups remained stable. Further, to confirm that rTsSp was a particular *T. spiralis* protein that had the immune protective effects in mouse colitis model, we observed the body weight change of mice from another recombinant protein (a newborn larvae-specific **c**ysteine **p**rotease **i**nhibitor from *Trichinella spiralis*, with a predicted molecular weight of 46.9 kDa, expressed in pET-22b (+) plasmid and purified in our lab) pre-administration-DSS group or protein non-derived from helminth pre-administration-DSS group, and found that there was no immune protective effect stemming from those proteins (**Supplementary Figure 3**). When the mice were sacrificed, we separated the colon tissues and measured the length, found that colons were thinner and shorter in mice treated with DSS alone, compared to those of control and rTsSp-immunized mice (**Figures 4B, C**). Histological examination of the colon showed that DSS administration (positive control) induced significant colonic damage as characterized by epithelial erosion, ulceration, crypt abscess, edema, loss of the mucus layer and substantial polymorphonuclear infiltrate into the lamina propria (**Figure 4D**), and rTsSp pre-immunization significantly prevented the damage in the colons produced by DSS. PBS control and rTsSp immunization alone did not result in microscopic damage of the colon (**Figure 4D**). These results demonstrate that rTsSp pre-immunization suppressed clinical signs in DSS-induced colitis in mice.

### Detection of Gut Macrophage by Immunofluorescence

**Figure 5:**
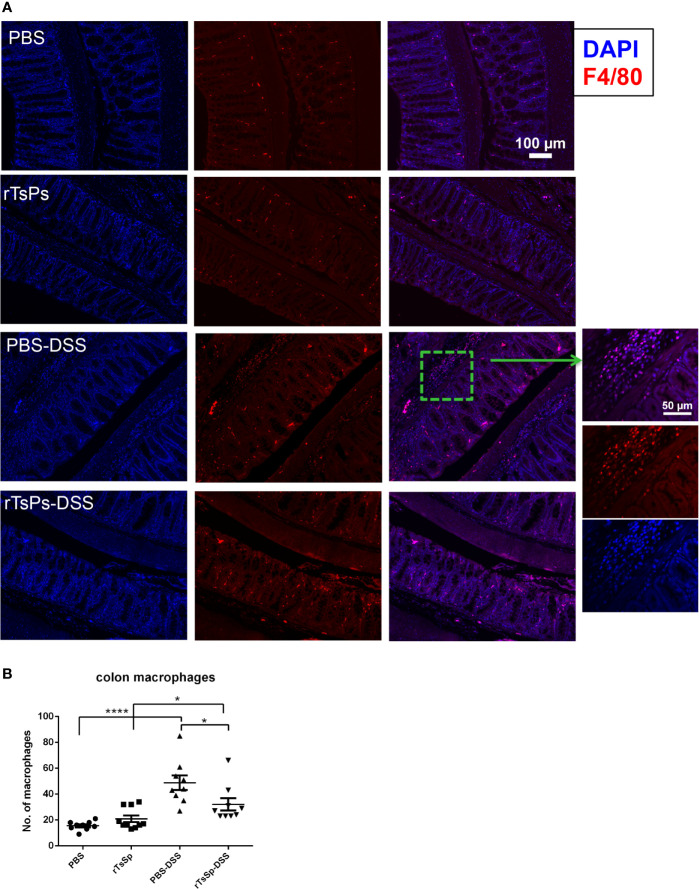
Pre immunization with rTsSp reduces macrophages recruitment to the DSS induced colitis of the colon. **(A)** Colon slides from PBS control, rTsSp pre immunization, DSS treatment, rTsSp pre immunization and DSS treatment mice were stained with anti-F4/80 antibody for macrophages. The number of F4/80^+^ cells **(B)** detected in each high power field was determined by counting 10 fields from each sample (samples from three mice per group were counted) using the Image J software. The data shown are means ± SEM. *P < 0.05, ****P < 0.0001.

F4/80 (indicates macrophages) was detected to assess the macrophages in colon tissues. F4/80 was labeled red, and nuclei were stained with DAPI. DSS treatment resulted in statistically significant quantities of F4/80 positive macrophages in both the absence and presence of the rTsSp pre immunization when compared to their respective controls, PBS or rTsSp pre immunization alone (**Figure 5A**). Meanwhile, there was a statistically significant increase in the quantity of macrophages in the colon of DSS-treated mice when compared to the mice in the concurrent presence of rTsSp pre immunization and DSS treatment (**Figure 5B**). These results indicated that pre immunization with rTsSp reduced macrophage recruitment to the DSS induced colitis of the colon.

### Immunization of the Mice With rTsSp Results in Suppression of the TNF-α Production by Peritoneal Macrophages

**Figure 6:**
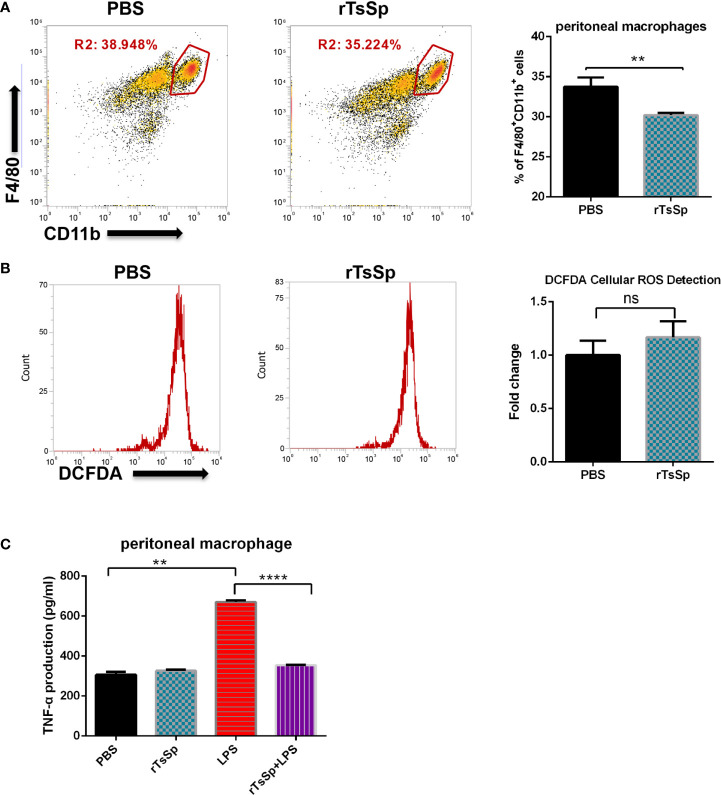
The effect of rTsSp on peritoneal macrophage population, ROS and TNF-α production. **(A)** Peritoneal macrophages were isolated from wild type and rTsSp pre-treated mice, identified with anti-F4/80 and CD11b mAb. **(B)** Intracellular ROS production was measured using the DCFDA. The stained samples were analyzed with BD FACSCanto. Results are expressed as fold change of mean fluorescent intensity after background subtraction between control and treated cells. **(C)** Immunization with rTsSp results in suppression of the TNF-α production by peritoneal macrophages. Peritoneal macrophages were isolated from PBS control and rTsSp-immunized mice and stimulated with LPS/PBS. The data shown are means ± SEM from one of three separate experiments performed showing similar results. **P < 0.01; ****P < 0.0001; ns, no significant.

To further analyze the possible association of the effect of rTsSp on mouse innate immune cells, we separated peritoneal macrophages, identified the cells, and analyzed the production of intracellular ROS and TNF-α. First, the mice were immunized with rTsSp (3 times, 7 days/time), PBS intraperitoneal injection was used as control. 21 days later, peritoneal macrophages were isolated from control and rTsSp-immunized mice. The cells were identified with anti-F4/80 and CD11b mAb, and the result showed that pre immunization with rTsSp resulted in a statistically relevant decrease in the population of F4/80^+^CD11b^+^ macrophages present in the peritoneal cavity when compared to WT control (**Figure 6A**). The DCFDA Cellular ROS Detection Assay Kit was used to detect the impact of rTsSp immunization on the intracellular ROS production of peritoneal macrophages. Cells were treated with PMA and stained in the DCFDA solution. The result showed that rTsSp pre immunization did not appear to have an impact on peritoneal macrophage ROS production (**Figure 6B**). For detecting TNF-α production, the cells were stimulated with LPS/PBS, after 5 h incubation, supernatants were collected and the levels of TNF-α production were analyzed. As shown in **Figure 6C**, intraperitoneal immunization with rTsSp resulted in suppression of TNF-α production. Significantly high levels of TNF-α production were detected from LPS stimulated peritoneal macrophage isolated from control mice, compared to the PBS stimulated, and both of PBS and LPS incubated peritoneal macrophages isolated from rTsSp immunized mice. These results demonstrated that rTsSp treatment reduced the population of F4/80^+^CD11b^+^ macrophages in the peritoneal cavity and suggested that rTsSp had the potential for modulating macrophage response.

### Prior rTsSp Treatment Has Differential Impact on Inflammatory Mediators

**Figure 7:**
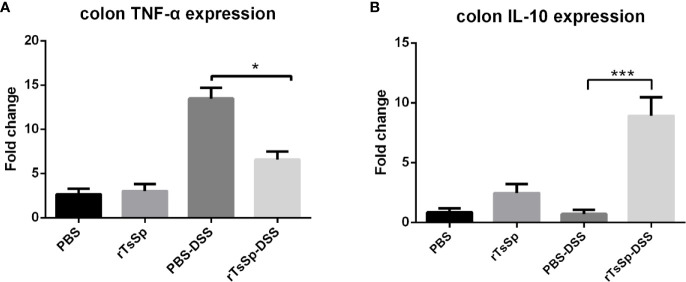
Prior rTsSp treatment has differential impact on inflammatory mediators. Colon tissues were collected. Total RNA was isolated. IL-10 and TNF*-α* expression was determined using RT-qPCR. Values are the relative expression compared to the PBS control group. The data shown are means ± SD from three independent experiments. *P < 0.05, ***P < 0.001.

To investigate the impact of rTsSp on the inflammatory response, the expression of IL-10 and TNF-α was detected using the real-time quantitative RT-PCR. The results showed that rTsSp pre-treatment reduced the colon tissue TNF-α expression of mice with DSS induced colitis (**Figure 7A**). In contrast, the rTsSp pre-treatment increased *IL-10* expression in the colon tissues of mice with DSS induced colitis (**Figure 7B**).

## Discussion

Millions of people worldwide currently suffer from parasitic helminth infections. The long-lasting infections of the helminth parasites result in the manipulation of the host immune responses causing serious health implications among people (26, 27). The immunomodulatory properties of *T. spiralis* have been validated in several models. Infection with living *T. spiralis* ameliorates experimental DSS-induced colitis or acetic acid-induced colitis in mice. *T. spiralis* muscle larval ES-treated dendritic cells ameliorates TNBS- induced colitis in mice (16).

Among various chemically induced colitis models, DSS- induced colitis model is widely used because of its simplicity (23) and most closely resembles human UC (28), which is clearly a global disease as its incidence is rising in nations around the world (29). Like UC, the mechanism by which DSS induces intestinal inflammation is also unclear. An important caveat pertaining to DSS colitis is that T and B cells are not required for development of colitis (23). Hence, the acute DSS colitis model is particularly useful when studying the contribution of the innate immune system to the development of intestinal inflammation.

Several helminthes, helminth extracts or helminth derived individual molecules, including recombinant proteins from *Ascaris lumbricoides* (30), *Wuchereria Bancroft* (31), *Brugia malayi* (32, 33), attenuate colitis in DSS models, by inducing anti-inflammatory cytokine-IL-10 and TGF-β expression and downregulating pro-inflammatory- IFN-γ, IL-6, IL-17 and TNF-α cytokines expression by colonic tissue/splenocytes. However, the cell types directly responsible for protective effects of the helminth protein in colitis have not been definitively resolved. In our present study, we expressed and purified the rTsSp and demonstrated that rTsSp has no toxic effects by cytotoxicity assay. Additionally *in vitro* TNF-α production by Raw 264.7 cells pre-incubated with rTsSp was reduced. We found that immunization of mice with rTsSp significantly attenuated macroscopical and histological features of DSS-induced colonic inflammation that were accompanied by decreased macrophage recruitment in the colon and reduced TNF-α mRNA levels in the colon; in contrast, the expression level of IL-10 increased in the colon tissue.

Macrophages are important in the host’s immunological and inflammatory responses. The intestine contains the largest pool of macrophages in the body, which are essential for maintaining mucosal homeostasis but are also involved in the pathology of IBD (34). The resident intestinal macrophages cannot be easily induced to mediate acute inflammatory responses. When there is perturbation of homeostasis, the composition of the intestinal macrophage pool changes considerably. In the mucosa of Crohn’s disease and ulcerative colitis patients, there is an increase in the mucosal macrophage population derived from circulating monocytes; these cells have the ability to produce many mediators that are important in the proinflammatory responses, such as IL-1, IL-6, TNF-α, reactive metabolites of oxygen and nitrogen and proteases, and the expression of iNOS (34–38). Colitis pathology has been shown to be ameliorated in mice deficient (39) or depleted (40) of these cells. Thus, the ability to reduce the monocyte/macrophage recruitment to the inflammation location is the protective mechanism of the rTsSp in the DSS-induced colitis model.

It is well known that the cytokine responses are the key pathophysiological elements that govern the inflammation of IBD. TNF-α is an important mediator of inflammation; it plays a critical role in the pathogenesis of IBD. Studies have shown that systemic TNF-α level is elevated in DSS-induced colitis (41, 42) and in IBD patients (43). Varol C. et al. (44) reported that TNF-α production by recruited macrophages appears to be central to disease progression, as the severity of DSS-induced colitis is reduced in mice in which Ly6C^hi^ monocytes are deficient in TNF-α production. And therapeutic monoclonal antibodies against TNF (anti-TNF therapy) are emerging as a common treatment for acute severe UC (45). In the present study, the TNF-α production by peritoneal macrophages of rTsSp immunized mice was reduced. IL-10 is a pre-dominantly anti-inflammatory cytokine with an essential role in maintaining gastrointestinal homeostasis, which is produced by hematopoietically derived cells, including T cells, B cells, dendritic cells, and macrophages (46), and potently inhibits production of most inducible chemokines that are involved in inflammation (47). Tomoyose et al. (48) reported that administration of systemic IL-10 ameliorates colitis in mice by inhibiting intestinal inflammation and suppressing TNF-α and IL-1β production. In our results, mice immunized with rTsSp produced high level of IL-10 in the presence of DSS administration. Toll-like receptors (TLRs) are essential components of the innate immune system. Almost all TLRs (except TLR3) signal *via* a MyD88-dependent signaling pathway (49). Previous research findings have shown that helminth and helminth-associated protein up-regulated the expression of TLR2 in mice (50, 51), and ligands recognized by TLR2 stimulated Th2 or Treg responses and IL-10 production (51–53). The rTsSp may carry the function of reducing the production of TNF-α and inducing the expression of IL-10 by stimulating the TLR2/MyD88 signaling pathway. However, our present data are not sufficient to confirm whether the induction by rTsSp is due to stimulation of TLR2 on APC, which will require further analysis.

In summary, our data indicate that immunization with the individual recombinant protein of *T. spiralis-* rTsSp ameliorates the DSS-induced colitis in mice by decreasing the infiltration of macrophages, reducing the production of TNF-α and inducing the expression of IL-10. These findings suggest that rTsSp may have therapeutic potential on colitis, and that immunotherapy with rTsSp represents an additional strategy to improve inflammatory processes of IBD.

## Conclusion

The unique helminth derived protein- rTsSp is able to ameliorate disease signs in an acute model of colitis *via* its immunomodulatory properties, which suggests that rTsSp have therapeutic potential on colitis, and immunotherapy with rTsSp represents an additional strategy to improve inflammatory processes of IBD.

## Data Availability Statement

The data sets presented in this study can be found in online repositories. The names of the repository/repositories and accession number(s) can be found below: https://www.ncbi.nlm.nih.gov/, AY491941.

## Ethics Statement

The animal study was reviewed and approved by The Life Science Ethics Committee of Zhengzhou University.

## Author Contributions

SL and RL performed experiments. SL, ZW, and C-WS designed experiments. SL and DK analyzed data and wrote the paper. ZW, C-WS, and DK participated in editing and provided conceptual advice. All authors contributed to the article and approved the submitted version.

## Funding

This work was supported by grants from National Key Research and Development Program of China (2017YFD0501302) and China Postdoctoral Science Foundation (2020M672287).

## Conflict of Interest

The authors declare that the research was conducted in the absence of any commercial or financial relationships that could be construed as a potential conflict of interest.

## Abbreviations

IBD, inflammatory bowel disease; rTsSp, recombinant *T. spiralis* newborn larvae-specific Serine protease; DSS, dextran sodium sulfate; MLN, mesenteric lymph nodes; TNF-α, tumor necrosis factor alpha; IL-10, interleukin-10; UC, ulcerative colitis; ROS, reactive oxygen species; PMA, phorbol 12-myristate 13-acetate; LPS, lipopolysaccharide; ELISA, enzyme-linked immunosorbent assay; PBS, phosphate-buffered saline; cDMEM, complete Dulbecco’s modified Eagle’s medium; DCFDA, 2′,7′-Dichlorofluorescin Diacetate; WT, wild type; DAPI, 4′, 6-diamidino- 2-phenylindole; PI, Propidium Iodide; FLICA, fluorochrome-labeled inhibitors of caspases.
