# Supplementary material for: Immune Protection of a Helminth Protein in the DSS-Induced Colitis Model in Mice
Source: Front Immunol. 2021 Apr 29;12:664998. doi: 10.3389/fimmu.2021.664998 (PMC8117093; doi:10.3389/fimmu.2021.664998)
Supplement: Supplementary file 1 [file DataSheet_1.docx]

**Immune protection of a helminth protein in the DSS-induced colitis model in mice**

Shao Rong Long^1, 2^, Ruo Dan Liu^1^, Deepak Vijaya Kumar^2^, Zhong Quan Wang^1^, Chien-Wen Su^2, *^


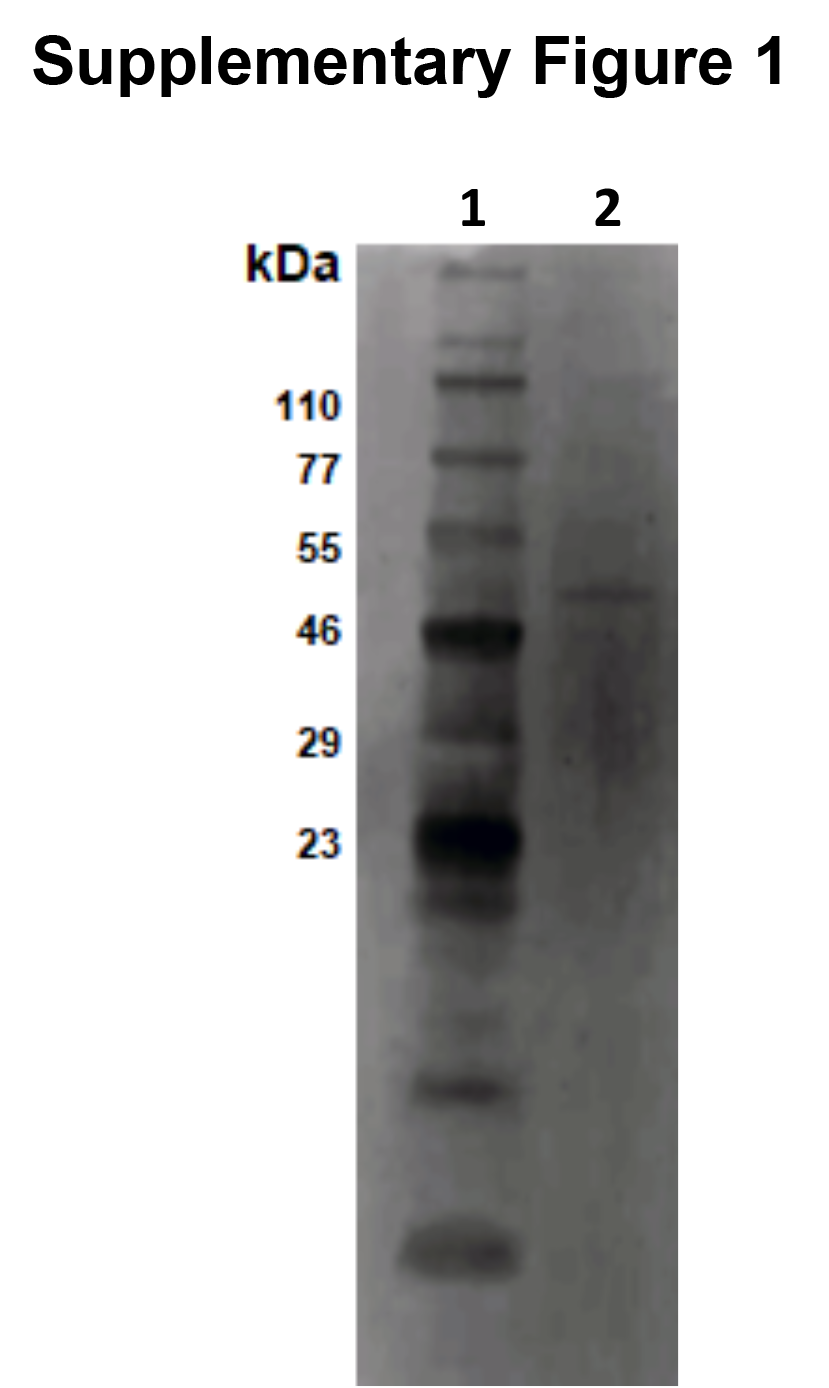


**SUPPLEMENTARY FIGURE 1 |** Silver stain analysis of rTsSp protein. rTsSp protein was separated on a NuPAGE® 4-12% Bis-Tris Gel and then stained with the ProteoSilver® Silver Stain Kit. Line 1: Marker STD diluted 1:4; Line 2: 1 ug of purified rTsSp protein


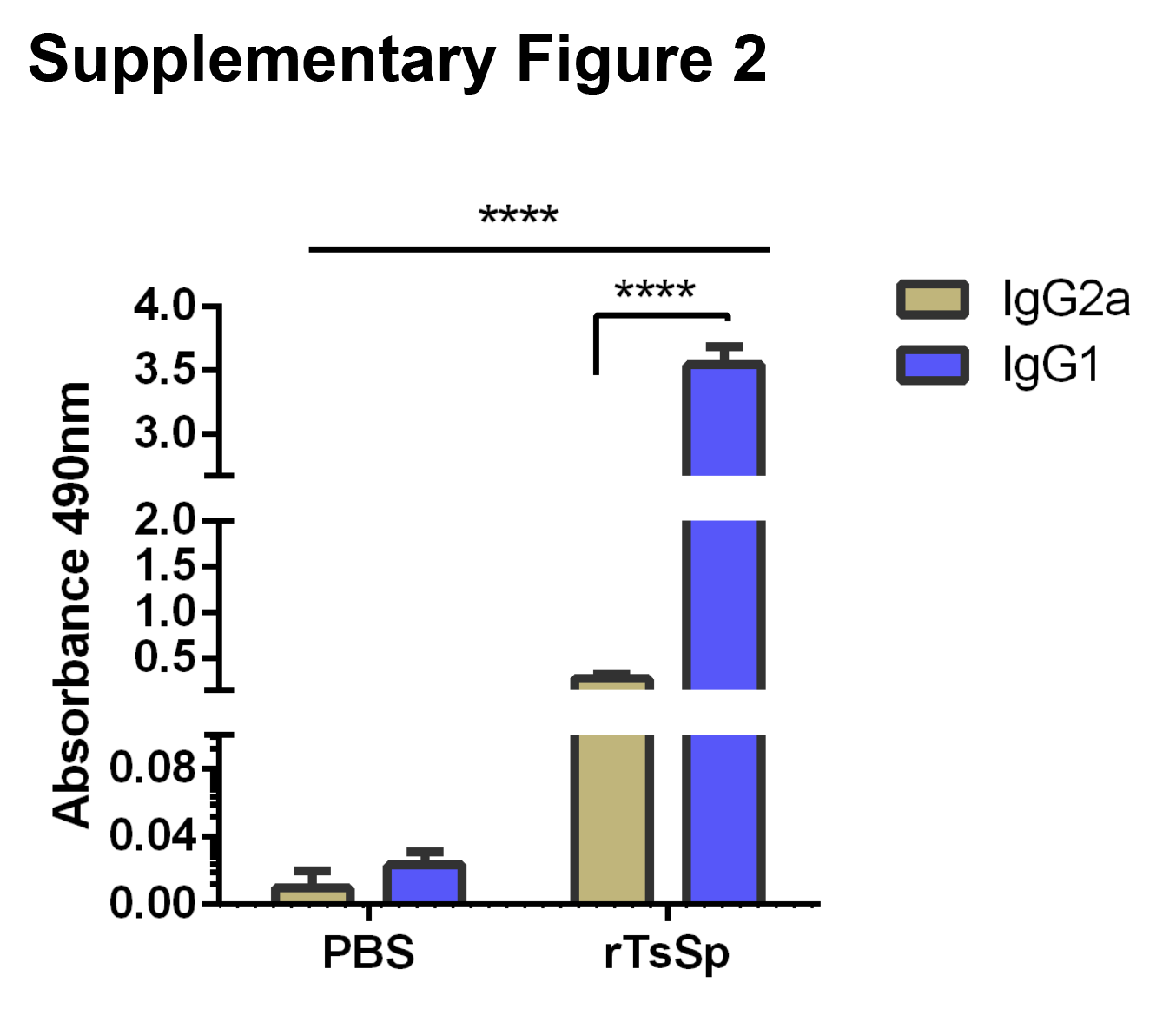


**SUPPLEMENTARY FIGURE 2 |** Analysis of antibody responses in mice. The anti-rTsSP specific IgG subclass (IgG1 and IgG2a) responses in the sera of immunized mice were detected by ELISA. The OD490 values shown for each group are the mean ±  SEM of the antibody levels from three separate experiments. *****P*<0.0001


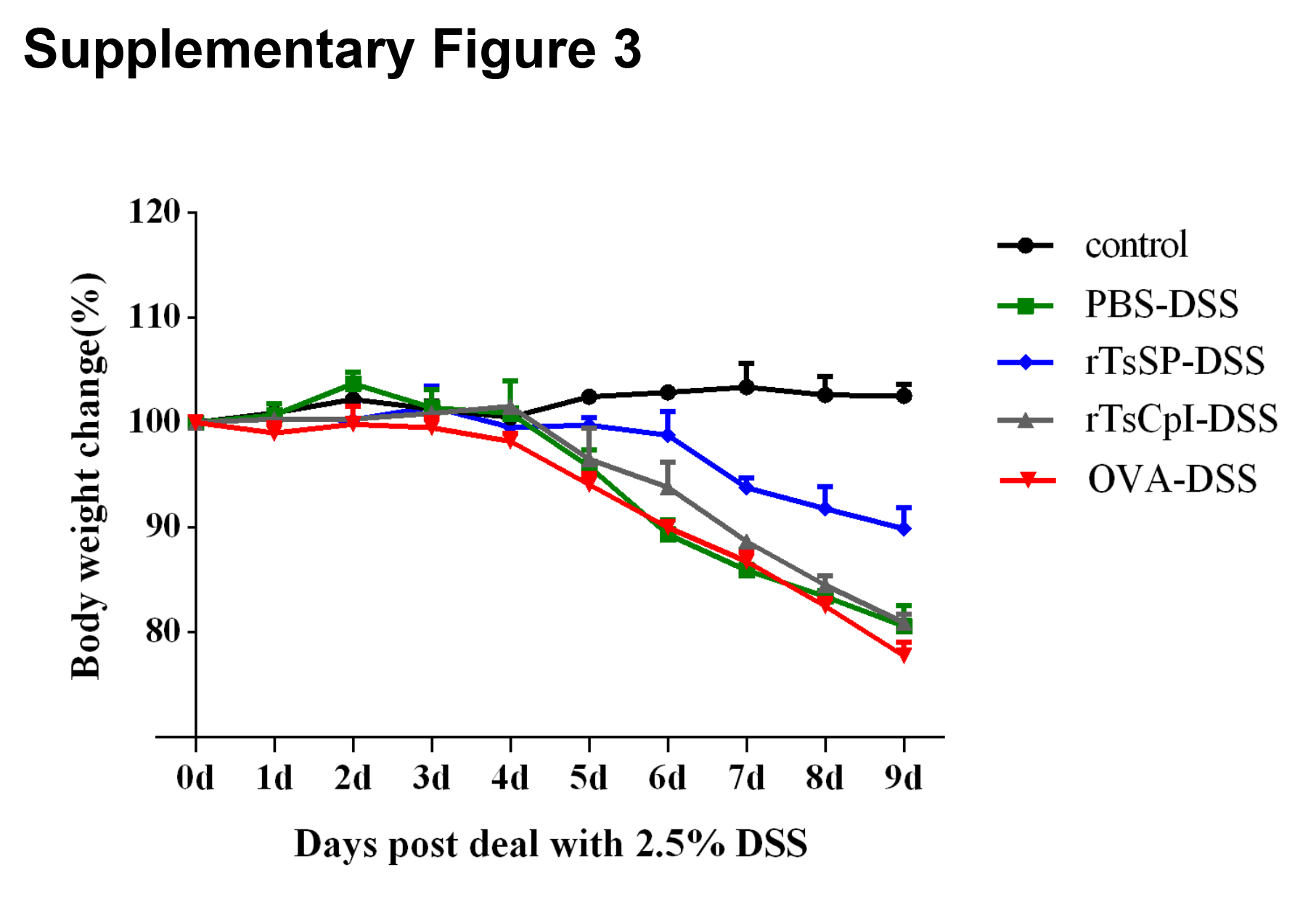


**SUPPLEMENTARY FIGURE 3 |** Protein other than rTsSp showing no effect on DSS induced colitis in mice. Mice were immunized and induced colitis as MATERIAL AND METHODS. Immunized proteins included rTsSp, rTsCpI (another recombinant protein- **r**ecombinant ***T****richinella* ***s****piralis* **c**ysteine **p**rotease **i**nhibitor, 46.9 kDa, purified in our lab) and OVA (ovalbumin, protein non-derived from helminth; purchased from Sigma). Recombinant plasmids of rTsSp, rTsCpI was provided by Prof. Mingyuan Liu from Jilin University. The data shown are means ± SEM from one of three separate experiments performed showing similar results.
